# The rule of four: anomalous distributions in the stoichiometries of inorganic compounds

**DOI:** 10.1038/s41524-024-01248-z

**Published:** 2024-04-12

**Authors:** Elena Gazzarrini, Rose K. Cersonsky, Marnik Bercx, Carl S. Adorf, Nicola Marzari

**Affiliations:** 1 Theory and Simulation of Materials (THEOS) and National Center for Computational Design and Discovery of Novel Materials (MARVEL), École Polytechnique Fédérale de Lausanne, CH-1015 Lausanne, Switzerland; 2 Department of Chemical and Biological Engineering, University of Wisconsin - Madison, Madison, WI USA

**Keywords:** Condensed-matter physics, Condensed-matter physics, Structure of solids and liquids

## Abstract

Why are materials with specific characteristics more abundant than others? This is a fundamental question in materials science and one that is traditionally difficult to tackle, given the vastness of compositional and configurational space. We highlight here the anomalous abundance of inorganic compounds whose primitive unit cell contains a number of atoms that is a multiple of four. This occurrence—named here the *rule of four*—has to our knowledge not previously been reported or studied. Here, we first highlight the rule’s existence, especially notable when restricting oneself to experimentally known compounds, and explore its possible relationship with established descriptors of crystal structures, from symmetries to energies. We then investigate this relative abundance by looking at structural descriptors, both of global (packing configurations) and local (the smooth overlap of atomic positions) nature. Contrary to intuition, the overabundance does not correlate with low-energy or high-symmetry structures; in fact, structures which obey the *rule of four* are characterized by low symmetries and loosely packed arrangements maximizing the free volume. We are able to correlate this abundance with local structural symmetries, and visualize the results using a hybrid supervised-unsupervised machine learning method.

## Introduction

Computational materials discovery is a fast-growing discipline leading to innovation in many fields. Within a specific technological sector (i.e., communications, renewable energies, medical), the choice of material is critical for the long-lasting success of the given product. Therefore, it is important—and of fundamental interest—to efficiently identify materials’ structural and energetic characteristics through materials’ data analysis to select structures for innovative applications. The emerging field of materials informatics has demonstrated its potential as a springboard for materials development, alongside first-principles techniques such as density-functional theory (DFT)^1,2^. The increase in computational power, together with large-scale experimental^3^ and computational high-throughput studies^4^, is paving the way for data-intensive, systematic approaches to classify materials’ features and to screen for optimal experimental candidates. In addition, the collection of statistical methods offered by machine learning (ML) has accelerated these efforts, both within fundamental and applied research^5–10^.

**Fig. 1:**
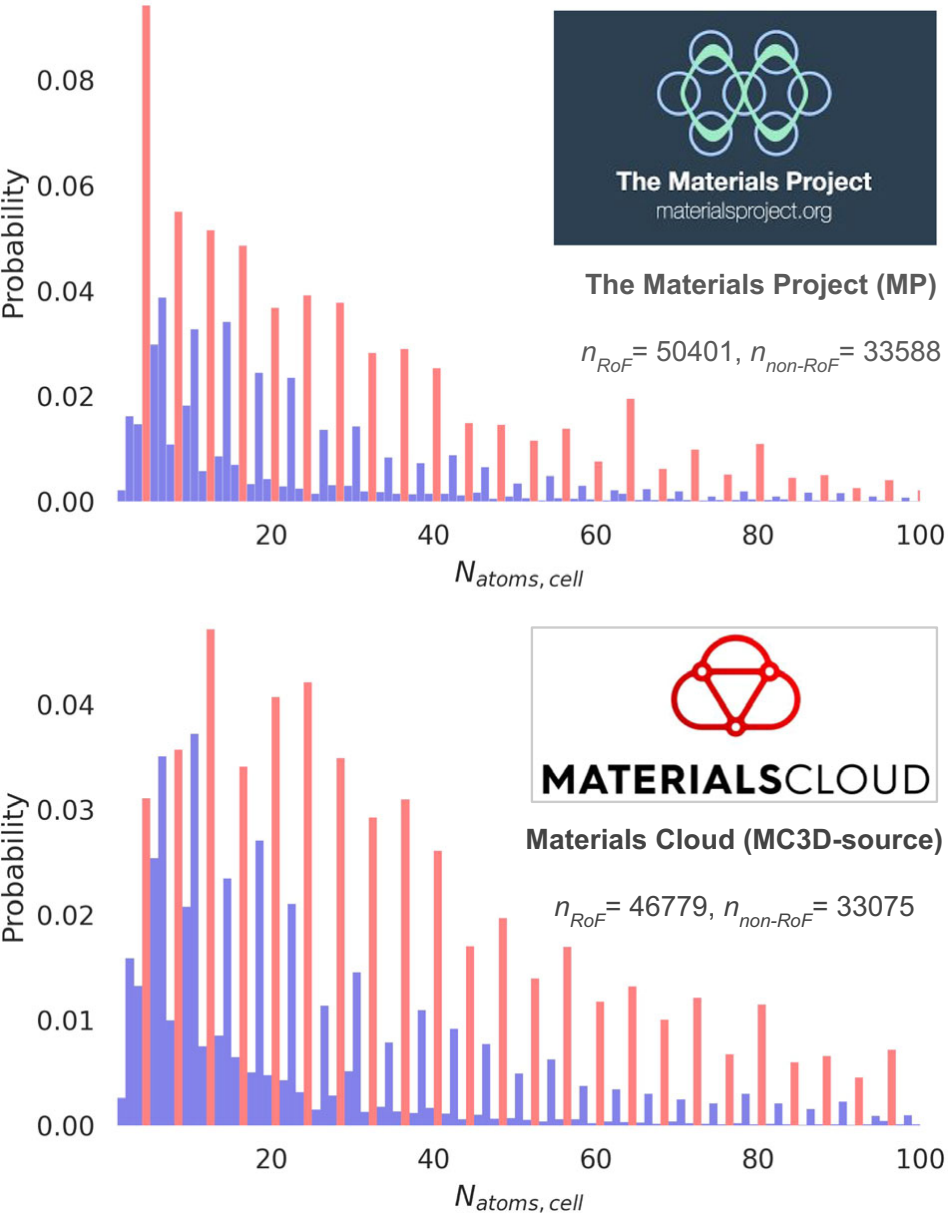
The rule of four. The two datasets (the Materials Project (MP)^11^ and the Materials Cloud 3-dimensional crystal structures ‘source’ database (MC3D-source)) contain a disproportionate amount (coloured in red) of compounds with a primitive unit cell containing multiples of 4 atoms. *n*_*RoF*_ characterises the number of structures in the datasets that obey the *rule of four*, while *n*_*non*−*RoF*_ the ones that do not. The distributions are normalised.

However, the success of these endeavours is ultimately limited by the quality and diversity of the data serving as the underlying data source. Understanding the space of materials spanned by a dataset is integral to data-driven materials searches or machine-learning workflows. Thus, when anomalous correlations arise in datasets, it is useful to understand and investigate the origins, and potential implications, of such peculiarities. We use here the name *rule of four* (RoF) to describe the unusually high relative abundance of structures with primitive unit cells containing a multiple of 4 atoms. This occurrence is explored within two different databases of inorganic crystal structures: the Materials Project (MP)^11^ database, which contains crystal structures that have been relaxed with first-principles calculations starting from experimental databases or from structure-prediction methods, and the Materials Cloud 3-dimensional crystal structures ‘source’ database (MC3D-source); this latter combines experimental structures from the crystallographic open database (COD)^12–15^, the inorganic crystal structures database (ICSD)^16^ and the materials platform for data science (MPDS). Note that for the ICSD and COD, occasionally some theoretically predicted structures can also be present, see section I in the supplementary information for more details. Figure 1 is a visual representation of this striking abundance, while Table 1 demonstrates the RoF by comparing the relative abundance of structures with primitive unit cells made up of multiple of 3, 4, 5, 6 and 7 atoms.

**Table 1:** Percentages of structures in the MP and MC3D-source databases whose primitive unit cells contain a number of atoms that is a multiple of the column header

multiple of	3	**4**	5	6	7
**Materials Project**	32.38	**60.01**	18.41	26.82	12.43
**MC3D-source**	36.57	**58.58**	20.89	30.99	12.51

The RoF emerges from the higher abundance of structures with a primitive unit cell containing a multiple of 4 atoms. Primitive unit cells with a number of atoms that is a multiple of two or more headers will contribute to each column; hence, the percentages will sum to > 100.

Within the context of this study, we will label a structure that belongs to the subset of structures with a unit cell size multiple of four as a RoF structure, and one that does not belong to the subset as a non-RoF structure. In Fig. 1 the *x* axis is capped at 100 atoms to best represent the RoF, as respectively 97.51% and 91.00% of structures in the MP and in the MC3D-source databases contain 100 atoms or less (the largest cell in the MP database contains 296 atoms, while the MC3D-source one contains 4986 atoms).

Before delving into an extensive analysis, we rule out that the RoF is simply an artefact of how structures are mathematically described, or of how this description is curated and processed for storage in the aforementioned databases (III A). We then decide to probe the RoF more deeply and attempt to understand its origins and impact. First, we examine the RoF with respect to traditional materials science metrics, including energies and symmetries, and uncover that the RoF is largely correlated with loosely-packed polyatomic systems (III B, III C). We then use symmetry-adapted machine learning techniques to relate the RoF to local atomic environments and determine that it has only little implications for formation energy (III D). We finally manage to correctly classify the RoF by only providing the algorithm with information on local structural symmetry rather than a global one (III D). Although we explored many meaningful avenues to rationalize the rule’s existence and emergence, a full explanation of the anomalous distribution is still missing. Since the most plausibile causes have been explored, the present work serves also as a reference for future research on the topic.

## Results

Within this study, we make sure that the data is sufficiently diverse for the training set to cover the whole design space^17^ by procuring the structural data from open and FAIR repositories^18–20^; the same analytical workflow is applied to two different databases of bulk, crystalline, stoichiometric compounds. One database is the Materials Project, which contained 83 989 data entries obtained via high-throughput DFT calculations as of 10/18/2018, corresponding to the mp all 20181018 dataset retrieved with the matminer.datasets module^21^. The other data source, the MC3D-source, contains 79 854 unique structures extracted from the MPDS, ICSD and COD, which have been curated via an AiiDA^22^ workflow, as explained in Section I of the SI.

### Primitive unit cell

**Fig. 2:**
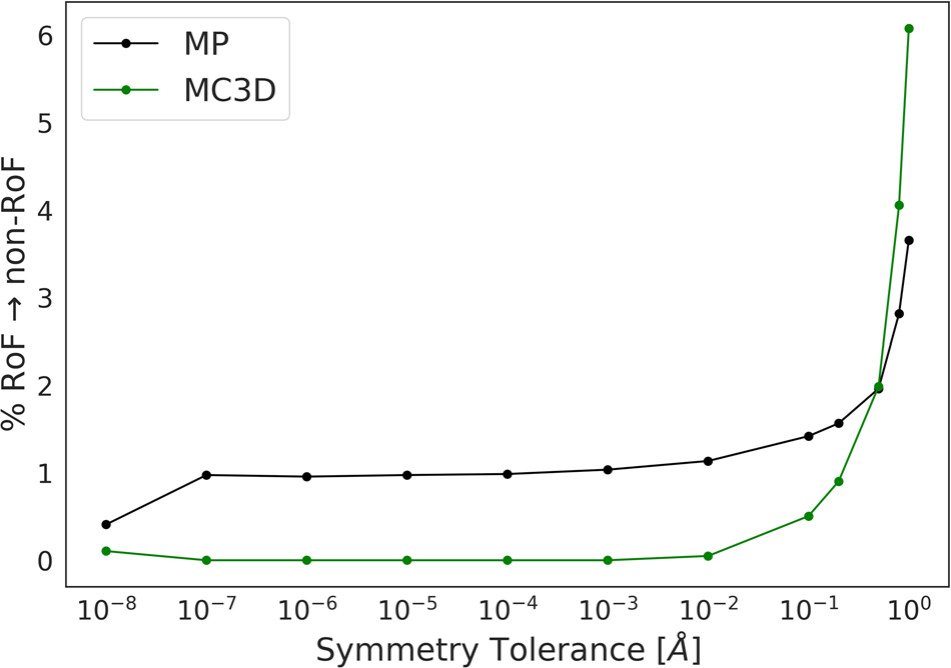
Percentage of RoF structures that become labelled non-RoF as a function of the symmetry tolerance parameter used for reduction to the primitive cell. The black and green lines correspond to structures in the MP and MC3D-source datasets, respectively. At typical symmetrization parameters, there is little to no change in the number of RoF structures (roughly 1% of RoF structures go to non-RoF). At larger symmetrization parameters (≈1 Å), this increases to roughly 6% based upon the large deviations allowed in considering sites as symmetrically equivalent.

When materials structure datasets are prepared, it is standard procedure to ‘primitivise’ unit cells, i.e., to reduce the unit cell to its minimum volume. As many conventional unit cells contain exactly four times the number of atoms that would be found in their respective primitive unit cell, it could be expected that misclassifying conventional unit cells as primitive ones could lead to an artificial emergence of the RoF. Both the MP and MC3D-source databases obtain the primitive unit cell using the spglib software^23^. When primitivizing the structure, one needs to set the symprec tolerance parameter, which allows for slight deviations in the atomic positions stemming from thermal motion or experimental noise. To rule out that the primitivization is the source of the emergence of the RoF, we show in Fig. 2 that changing the symprec (1E-8 to 1E-1Å) parameter has little effect on the RoF distribution, converting around 1% of RoF structures into non-RoF ones. It is only when one increases the symprec to unreasonably large values (close to 1 Å) that the slope changes—this is expected, as using such a large tolerance effectively considers sites with the same element that should be different as identical, producing primitive unit cells with a reduced number of sites, but which no longer correctly describe the structure. Encouraged by these results, we proceed with a more extensive analysis.

### Formation energy

We first test whether the RoF is correlated with stability with respect to elemental phases, as this would provide a straightforward explanation for the phenomenon. To test this assumption, we analyze the information contained in the MP dataset, namely the formation energy per atom within each compound. This is the energy of the compound with respect to standard states (elements), normalized per atom. For example, for Fe2O3 the formation energy is [*E*(*Fe*_2_*O*_3_)–2*E*(*Fe*)–(3*/*2)*E*(*O*_2_)]*/*5. It is computed at a temperature of 0 K and a pressure of 0 atm. This quantity is often a good approximation for formation enthalpy at ambient conditions, where a negative formation energy implies stability with respect to elemental compounds.

**Fig. 3:**
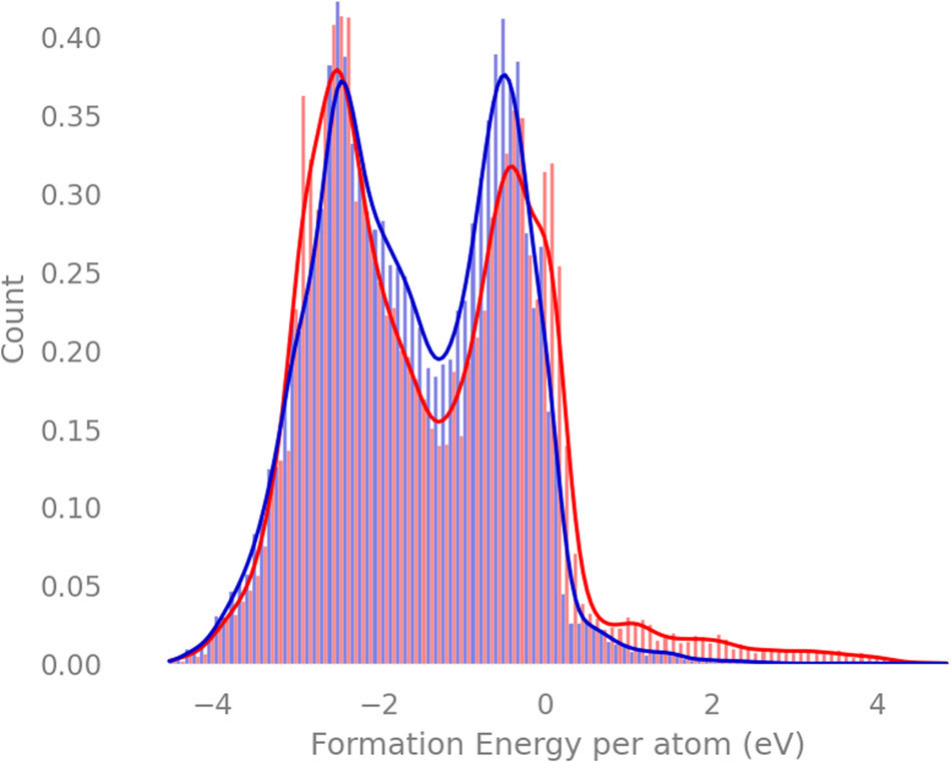
Distribution of formation energies. Normalised distribution of formation energies for the 83 989 compounds from the Materials Project, normalized for each subgroup. RoF compounds are coloured in red and non-RoF are coloured in blue.

Our initial results provide no evidence of a correlation between RoF compounds and their formation energy, as shown in Fig. 3. Nevertheless, it does appear that structures obeying the RoF have a longer positive tail of large formation energies, seen towards the bottom right of the figure.

However, this result can be misleading—it does not take into consideration the large variance in structural composition across the database—and we must aim to compare the energies of similar structures within the RoF and non-RoF subsets, as we will do in later sections.

### Correlation with symmetry descriptors

**Fig. 4:**
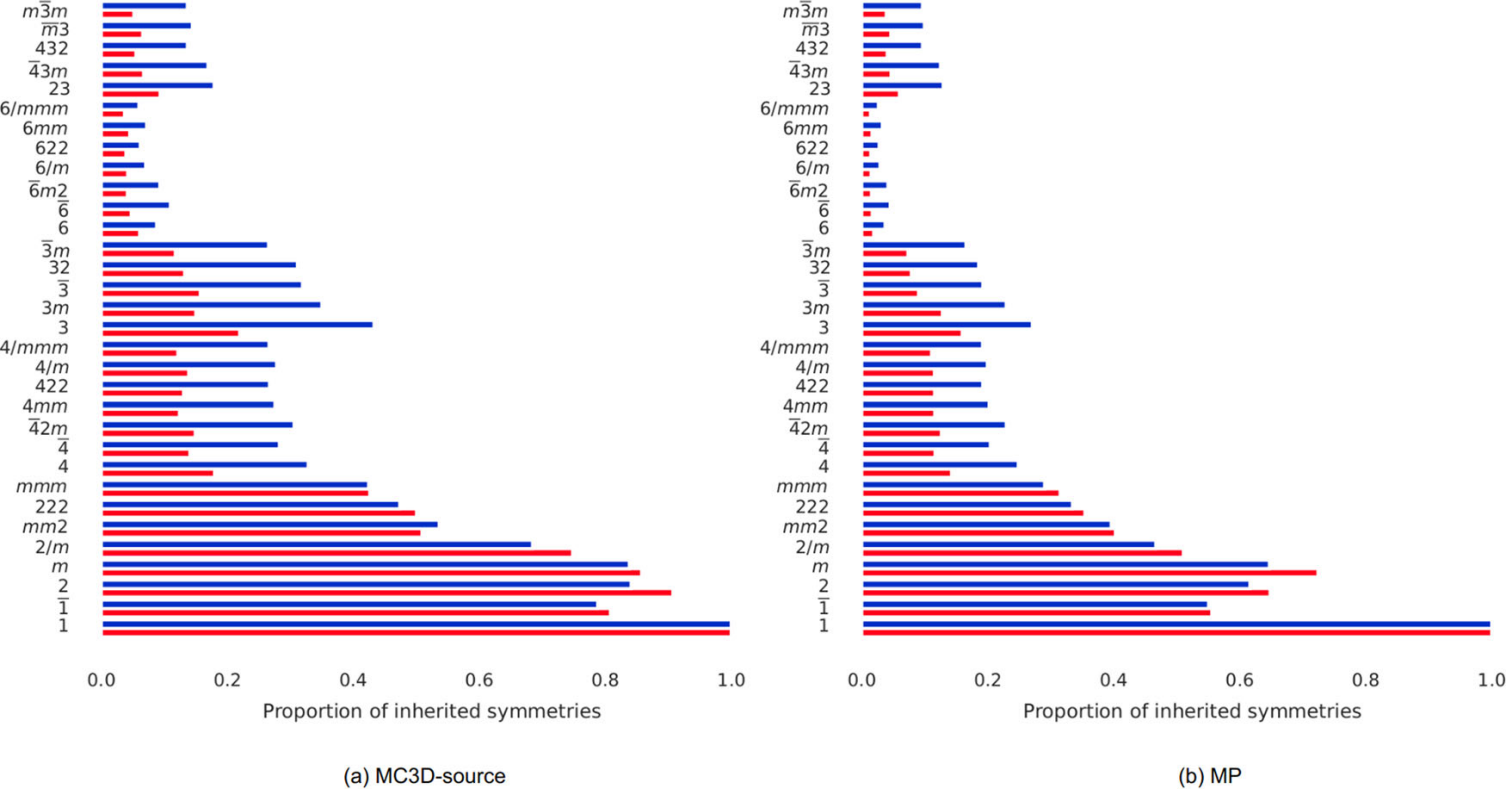
Point groups analysis. Proportion of structures in both databases (**a**) MC3D-source and (**b**) MP that belong to each point group represented on the *y* axis, counted based on their inherited symmetries. RoF compounds are coloured in red, while non-RoF ones in blue.

The crystal symmetries of compounds—defined by the set of symmetry operations that, when performed, leave the structure unchanged—are captured in crystals by their space groups and point groups. Higher symmetry space groups inherit the symmetry operations of their ‘parent’ point groups; for example, cubic space groups inherit the one-fold, two-fold, and four-fold rotational symmetries (for the interested reader, the concept of inherited symmetry is enumerated nicely in Fig. 1.5 of the book chapter by Hestenes^24^). Figure 4 shows histograms of inherited symmetries and their relative abundance within each of the two sets (RoF in red and non-RoF in blue). The point groups are ordered from the ones with the least number of symmetry operations (bottom) to the highest order ones (top). Symmetry groups that are equally represented in both sets (i.e. 1-rotation, since all compounds are invariant to the simplest symmetry) have tails of equal length, whereas symmetries seen in a larger percentage of RoF structures have a red tail to the right of the histogram.

From Fig. 4, the relative abundance of non-RoF structures in the high symmetry point groups emerges, while on the contrary most RoF structures in both databases are grouped in the lowest symmetry point groups (2, *m*, 2*/m*, *mm*2, 222 and *mmm*), which generally contain a relative abundance of them apart from one exception (the MC3D-source presents. a slightly higher relative abundance of non-RoF structures in the *mm*2 point group). This analysis shows how 4-fold symmetry is *not* a determining descriptor to classify the phenomenon.

**Fig. 5:**
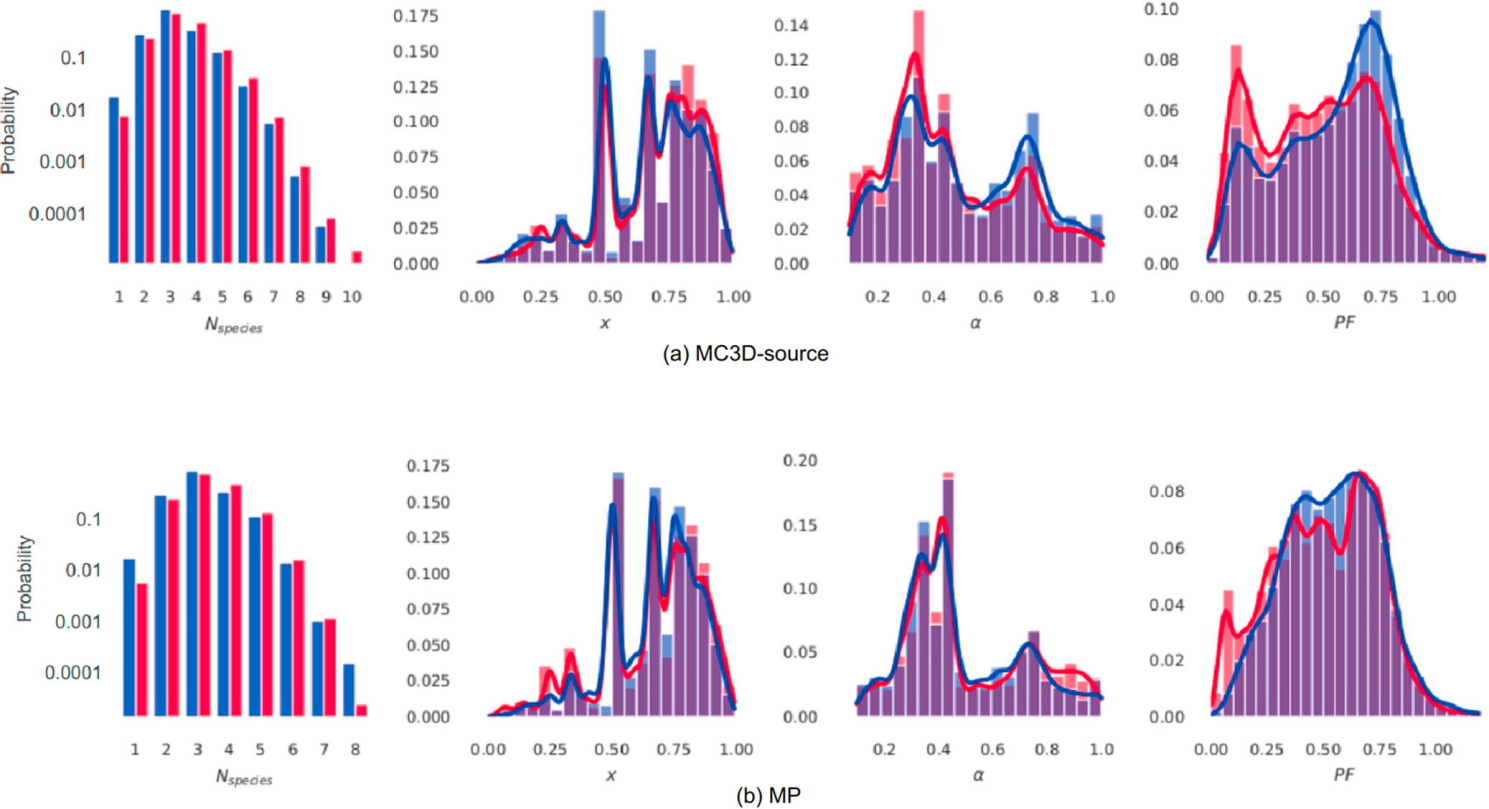
Distribution of geometric properties. Different geometric properties of each compound are analysed for the (**a**) MC3D-source and (b) MP databases. From left to right, the plots represent the normalised distribution of the number of elemental species (*N*_*species*_), the relative abundance of small (*N*_*S*_) to large (*N*_*L*_) atomic radii (*x*), the ratio between smallest (*R*_*S*_) and largest (*R*_*L*_) atomic radii (*α*) and the packing fraction (*PF*) for compounds with a unit cell size between 0 and 100 atoms. All of the results are plotted for the two sets, RoF (red) and non-RoF (blue), with the probability normalized to each set.

The the lack of higher symmetry groups in RoF compounds could be correlated by a heterogeneous composition of atoms; this heterogeneity can be quantified by counting the number of atomic species (*N*_*species*_) (first column of Fig. 5, in logarithmic scale) composing the structures: from this analysis we see that RoF materials are mostly composed of 4 or more elements (statistics start being less reliable after *N*_*species*_ = 8), while non-RoF structures present a larger abundance of simpler composition, containing more often 1, 2, or 3 elements. When looking at chemical composition, the hypothesis of the RoF emerging from signatures associated to a specific element have been ruled out. For example, when selecting from both datasets only structures containing Si (the first direct candidate having a typical coordination number of 4), the RoF still emerges with a probability of 59.07% for the MP dataset and 57.32% for the MC3D-source one. These statistics are not sufficiently divergent from the results in Table 1.

Another property that emerges from our analysis and is more evident in the MP dataset (second column of Fig. 5(b)) is the relative abundance of smaller atomic radii within RoF compounds, as often defined by the parameter $x=\frac{{N}_{s}}{{N}_{s}+{N}_{L}}$^25^, where *N*_*S*_ and *N*_*L*_ are the counts, in a given structure, of the smallest and largest radii respectively. When considering this parameter, we focus our attention on the MC3D-source dataset, which contains bigger and more complex structures, where the divergence between the smallest and largest atoms is more considerable. In fact, the first peak in Fig. 1 for the MP is very large, hinting at the implicit bias of computational studies, where larger structures are often avoided based upon computational cost of calculations. The abundance of small atomic radii in RoF compounds of the MC3D-source dataset (higher *x* parameter) partly explains the lower symmetries that characterise them, as more atoms will be inserted as ‘interstitial’ elements in a given structure, characterising the ‘imperfections’ that eventually contribute in lowering the overall structural symmetry of point groups analyzed in Fig. 4.

In general, the symmetry type of atomic crystal systems is strictly linked to packing mechanisms^26–28^. While the mathematical problem of sphere packing is not hard to pose (Kepler conjecture), it was historically difficult to prove^29^, and the complexity of its solution rises exponentially with polydispersity^30^.

Despite this, a qualitative analysis of RoF configurations shows that they contain chemical elements whose size variance is greater compared to the variance in the non-RoF population.

This size variance is quantified by the parameter $\alpha =\frac{{R}_{S}}{{R}_{L}}$ (where *R*_*L*_ is the radius of the largest atomic radius and *R*_*S*_ of the smallest one), namely the ratio between the smallest and the biggest atomic radii within each compound (third column of Fig. 5). For the same reason as above, in the context of this parameter the results of the MC3D-source dataset are considered more relevant. RoF compounds from the MC3D-source exhibit a greater standard deviation between largest and smallest atoms, with the *α* parameter presenting a peak at around 0.35; this finding suggests the presence of very small radii filling the interstitial spaces, which contribute to keeping the symmetry of RoF compounds low, as was previously highlighted by the analysis of the *x* paramter.

However, there is no overarching evidence in the distributions of the *x* and *α* parameters that allows us to confirm a correlation between the emergence of the RoF and the abundance of ‘intestitial’ elements.

The packing fraction (PF), defined as as ${PF}=\frac{{V}_{{tot},{atoms}}}{{V}_{{cell}}}$ (where *V*_*tot,atoms*_ is the total volume of all atoms composing the structure, and *V*_*cell*_ is the unit cell’s volume) is another related property of sphere packing^26,27^. This quantity is noticeably lower (with peaks at values around 0.1–0.2) for RoF structures, as can be seen in the last column of Fig. 5a, b, pointing away from packing arguments as the cause of this database anomaly. The sharp red peaks in *PF* might characterise disordered compounds such as porous materials, which have been determined to be outliers for the MC3D-source dataset.

### Employing symmetry-adapted descriptors for further insight

Up until this point we have employed classical techniques for analyzing crystal structures; these have offered little to no insights on the origins of the RoF anomalous distribution, but have allowed us to exclude packing arguments and symmetrical global descriptors as features that make the RoF emerge. Here, we therefore turn to more modern data- driven techniques. In the field of atomistic modelling, it has been common, albeit non-trivial, to represent crystal structures through symmetrized density correlations^9,31,32^ in order to predict broad swaths of materials properties. Here, we represent the compounds using the Smooth Overlap of Atomic Positions (SOAP)^31^, a popular ML representation for structure-energy relations that contains information on the average three-body local environment for atomic arrangements. SOAP vectors provide an avenue for a statistical analysis on local environments, offering a robust framework through which we can explore and visualize the chemical and configuration space of the materials studied^33^. We use two parameterizations of SOAP vectors, detailed in Section II of the Supplementary Information: one that uses separate channels to represent different chemical species and another that ignores the chemical identities in order to highlight the geometry of the local symmetry. The former, from hereon called the *species-tagged* representation, is necessary in energetic analysis, as similar geometry symmetries can correspond to wildly different energetics given the elements present; however, this representation is computationally cumbersome (roughly 100 000 sparse features for each compound, from which we take a diverse subset of 2 000 features). Thus, in later analyses where the chemical identities play a smaller role, it is beneficial and conceptually more straightforward to use the more lightweight, latter representation (roughly 80 features for each compound), hereon called the *species-invariant* representation.

**Fig. 6:**
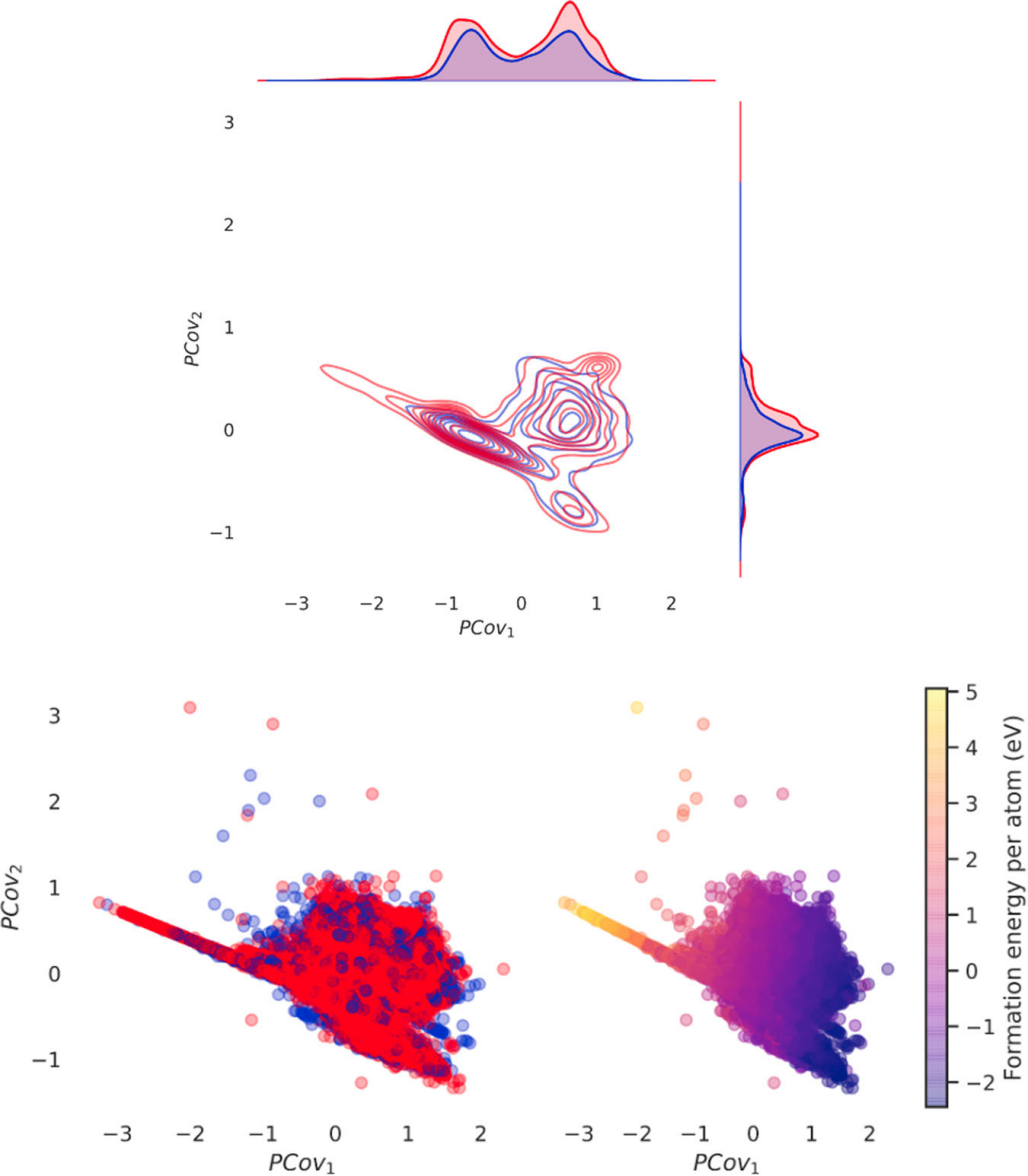
PCovR representation of the MP dataset with a mixing parameter of *β* = 0.5. The model is regressed on the formation energy per atom. The three plots contain the same data, represented on the top through a kernel density probability distribution (the RoF subset is coloured in red and the non-RoF one in blue), coloured according to the subset classification (lower left) and according to the formation energy per atom (lower right). The plot on the top is generated with the seaborn.kdeplot() function.

Earlier, we noted that simply presenting a histogram of RoF and non-RoF energetics did not provide any specific understanding of the RoF; it might be more insightful to compare the energies of chemically similar structures. To determine whether the RoF structures exhibit lower energy than structurally-similar non-RoF ones, we use Principal Covariates Regression (PCovR)^34,35^, a ML method which constructs a latent space projection to explore the correlation between stability and local symmetries within the dataset by expanding regression models to incorporate information on the structure of the input data, as implemented in the scikit-matter library^36,37^. In this mixing model, the projection is weighted towards the property of interest using a mixing parameter (of which a more extensive explanation is given in Supplementary Fig. 1), and, where the input linearly correlates with the target property, the resulting embedding will reflect this property along the first component, with subsequent components representing orthogonal dimensions in structure space. In our case the PCovR is always trained on the species-tagged SOAP vectors and their formation energies. We plot the first two principal covariates in Fig. 6. The first principal covariate is strongly correlated to the energetic descriptor, as can be seen in Fig. 6, where in the lower plots we have coloured each point in the projection by their RoF classification (left) and formation energy (right). However, the second covariate (and all significant subsequent covariates, see Supplementary Figs. 2-4) fail to separate the datasets into two distinct populations corresponding to this phenomenom. This implies that for structurally similar compounds, there is no significant difference in energy between RoF and non-RoF samples. We also see little difference in the spread of RoF versus non-RoF structures in the latent space, as shown by the kernel density probability map in the upper panel of Fig. 6. Further principal covariates for the same PCovR representation are plotted in Supplementary Figs. 2-4, as well as other relevant energetic descriptors (the energy above the convex hull energy, i.e., the envelope connecting the lowest energy compounds in the chemical space, and the band gap energy), in order to show how these targets yield similar results. Thus the RoF is neither correlated with the energetics, nor are RoF lower in formation energy when compared to chemically similar non-RoF ones.

**Fig. 7:**
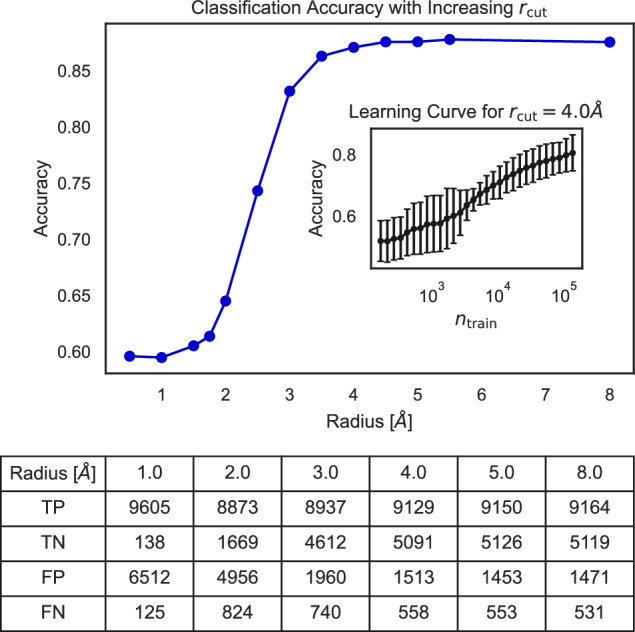
Random forest classification on local symmetries. Here we use the species-invariant 3-body SOAP vectors to build a random forest ensemble classifier. Test set accuracy, represented on the *y* axis, saturates at ~4.0 A, with little additional gain at larger cutoff radii. Below the figure we show the table of true positive (TP), true negative (TN), false positive (FP), and false negative (FN) results, showing that the classifier is unable to differentiate RoF and non-RoF structures at lower cutoff radii, leading to a high false positive (FP) rate. Inset in the upper figure is a learning curve for a cutoff radius of 4.0 Å, which shows a positive learning rate, albeit no saturation, an indication that secondary effects beyond the local environments play a role (or, more unlikely, that the dataset is not sufficiently large).

The linear correlation between the average local symmetries and the RoF is not particularly strong (a logistic regression on the SOAP vectors results in an accuracy on the order of 0.6, as listed in Table 1 of the SI); thus, we turn to non-linear classifications to understand if the RoF is potentially correlated with these local neighbourhoods. We ignore the species information to focus solely on the average local symmetries. We build a Random Forest (RF) classification^38^ on both datasets, first varying the interaction cutoff that defines the local environment (see Fig. 7). We see a plateau in accuracy on the test set at 87% as we consider local environments of 4.0 Å, suggesting that differentiating local symmetries occur within the first two neighbour shells, also supported by the high false positive (FP) rate at small cutoff radii. From the learning curve on the 4.0 Å escriptors (inset), we see that the classification has a positive learning rate, although shows little saturation despite the large training set. This result implies that local features are sufficient for the ML model to pick up the complexity of the datasets and to predict with good probability the correct classification. We report the accuracy on the test set achieved by other classification algorithms in Section V of the SI. To visualise the stoichiometry of materials falling into one of the two categories, the interested reader is referred to our Materials Cloud Archive^39^ entry, from which a json.gz file for each database can be downloaded. This can be uploaded on the Chemiscope^40^ web interface to visualise a 2D plot of the first principal covariates. The chemical composition of each dataset structure can be inspected by clicking on the dots composing the scatter plot. The colouring can be done according to different parameters, of which the most insightful one is the classification outcome.

## Discussion

Through an extensive investigation, in this work we highlight and analyze the anomalous abundance of inorganic compounds whose primitive unit cell contains a number of atoms that is a multiple of four, a property that we name *rule of four* (RoF) and that is observed in both experimental and DFT-generated structure databases. Here, we:

- highlight the rule’s existence, especially notable when restricting oneself to mostly experimentally known compounds;
- explore its possible relationship with established energetic descriptors, namely formation energies, and utilise hybrid ML methods combining regression and principal component analysis to surprisingly rule out the possibility that the relative abundance has the (expected) effect of stabilising compounds, bringing them to a lower energy state;
- conclude, through a global structural composition analysis of point groups and packing fractions, that the overabun- dance does not either correlate with high-symmetry structures, but rather to low symmetries and loosely packed arrangements maximising the free volume;
- predict, with an accuracy of 87% the association to the rule of four of a compound by providing a random forest classification algorithm with local structural descriptors (the smooth overlap of atomic positions) only, eventually highlighting the importance of local symmetry rather than global one for the emergence of the *rule of four*.

This analysis constitutes a valuable reference for further systematic studies targeting the classification of materials’ features with specific ML approaches in order to screen for optimal experimental candidates. Moreover, the study provides a starting point for future investigations on the rule’s emergence, given that a fully satisfactory explanation of such anomalous distribution is as yet lacking.

## Methods

### Reduction to the primitive cell

All the structures in both databases are reduced to the primitive cell using the find primitive function of the spglib^23^ package, varying the symprec value in the range of 1*E* − 8 to 1 Å.

### Scalar global descriptors

The symmetry of compounds is investigated by looking at space groups and point groups. subgroup of symmetry operations over which the space group is invariant. With a total number of 32 point groups, it is easier to convey the symmetric properties of the vast variety of compounds via their point group rather than their space groups; while space groups uniquely identify geometric properties, point groups identify symmetry classes and reduce the parameter space to a lower degree when investigating the symmetries of all compounds. The point groups are calculated through the spglib^23^ and seekpath^41^ packages for the MC3D-source database, while we used the SymmetryAnalyzer pymatgen module—which also relies on the spglib package developed by Togo and Tanaka^23^—to find the symmetry operators and point groups for the MP dataset, at a symprec of 0.3 Å. As concerns packing mechanisms, we extend the conventions employed by Hopkins^25^ to *n*-elements packing and employ the *α*, *PF* and *x* parameters. In structures with FCC and HCP symmetry, the maximum packing fraction is 0.74. *α* = 1 denotes unary compound. Conversely, when *α* 0 the compounds contain elements whose atomic radii distribution presents a wider spread.

### Local symmetry descriptors and ML pipeline

We adopt the following ML pipeline to study local symmetries and energetic effects. First, the atomic *representation* of each compound is obtained with SOAP vectors (see section II of the Supplementary Information), computed with the librascal library^33^. The SOAP features are then averaged within each compound, and the representations from the two datasets are normalised simultaneously. We then select a diverse subset of 2 000 features through Furthest Point Sampling (FPS) algorithm^36,37,42,43^, efficiently reducing the dataset size without losing important information. For Sec.III D, we perform a linear ridge regression with 4-fold cross-validation—which optimises the regularisation parameter to prevent overfitting—on the formation energies data retrieved from the MP database to ascertain the accuracy of the model. Table 2 illustrates the RMSE and the uncertainty in units of eV of the predicted energetic quantities.

**Table 2:** RMSE and uncertainty in units on the predicted energetic quantities for the MP database

Predicted quantity	RMSE	Uncertainty
Formation Energy per atom (eV)	0.0530	0.4002 eV
Energy above Convex Hull per atom (eV)	0.2938	4.0006 eV
Band Gap Energy with PBE- DFT functional (eV)	0.3097	3.6560 eV

The ML algorithm is a LRR with a 4-fold cross-validation. We report the formation energy per atom, the energy above the convex hull and the band gap energy.

Compared to results in the literature, which achieve an accuracy in formation energy prediction of 0.173 eV (Automatminer^44^) and 0.0332 eV (Crystal Graph Convolutional Neural Networks^45^), the accuracy of 0.4002 eV is sufficient for this study, since the aim of our study is not to find the most efficient way to predict energies, but rather to provide a sufficient regression prediction to employ in PCovR analysis. We use the species-invariant SOAP vectors to *classify* the RoF phe- nomenon using scikit-learn’s^46^ RandomForestClassifier algorithm^47^, which accepts binary labels as target properties (RoF or non-RoF) and outputs a probability between 0 and 1 for each compound to fall into the RoF subset. Training and testing set constitute respectively 90 and 10% of the whole dataset. Our random forest classification comprises 100 random decision trees. This classifier performs better in our case compared to Support Vector Machine (SVM) and Logistic Regression (LR) classifiers, signifying a need for a stochastic model.

## Supplementary information

The rule of four: anomalous distributions in the stoichiometries of inorganic compounds—Supporting Information

## Data Availability

The full dataset employed for the analysis can be downloaded from the Materials Cloud Archive^39^, where the MC3D- source data is only provided in SOAP format as the experimental structures can not be released due to licensing constraints. Its DFT–relaxed counterpart is available at: https://archive.materialscloud.org/record/2022.38. Instead, we provide the full list of structure IDs for each database, including the version of the database upon the time of extraction.

## Code availability

The codes to reproduce the results and figures can be found at: https://github.com/epfl-theos/r4-project. As the MC3D- source structure data cannot be made publicly available due to licensing contraints, the repository contains example data from a reduced random subset of the publicly available MP dataset in order to test run a preliminary analysis.
