# Supplementary material for: The rule of four: anomalous distributions in the stoichiometries of inorganic compounds
Source: NPJ Comput Mater. 2024 Apr 12;10(1):73. doi: 10.1038/s41524-024-01248-z (PMC11090804; doi:10.1038/s41524-024-01248-z)
Supplement: Supplementary file 1 — The rule of four: anomalous distributions in the stoichiometries of inorganic compounds—Supporting Information [file 41524_2024_1248_MOESM1_ESM.pdf]

Supplementary Information. The rule of four: anomalous distributions in the stoichiometries of inorganic compounds

Elena Gazzarrini[1], Rose K. Cersonsky[2], Marnik Bercx[1], Carl S. Adorf[1], and Nicola Marzari[1]

[1] *Theory and Simulation of Materials (THEOS) and National Center for Computational Design and Discovery of Novel Materials (MARVEL), École Polytechnique Fédérale de Lausanne, CH-1015 Lausanne, Switzerland*
[2] *Department of Chemical and Biological Engineering, University of Wisconsin - Madison, Madison, Wisconsin, USA*

I. Materials Databases

In this section we introduce the two datasets employed in the study and explain how the raw data is obtained.

The Materials Cloud 3-dimensional crystals Database - MC3D

The MC3D [1] is a database of structures optimized with the Quantum ESPRESSO code [2, 3] using fully-automated workflows developed in AiiDA [4, 5]. The starting set of structures for the geometry optimization is obtained from the COD [6], the ICSD [7] and the MPDS [8] databases. Each CIF file is parsed via an AiiDA workflow that removes unnecessary tags, performs minor corrections to the syntax, and parses the contents to extract the corresponding structure. The parsed structures are subsequently normalized and primitivized using SeeK-path [9], and a uniqueness analysis is performed to remove duplicate structures. Finally, hydrogen-containing structures from the COD are removed due to the prevalence of molecular crystals in this database, and any structure containing an actinide is also excluded from the database. The resulting 79 854 structures *before geometry optimization* are labelled as MC3D-source and used for the analysis in this paper. In this early version of the MC3D-source database, most (63 093) of the structures came from the MPDS, 13 798 were obtained from the ICSD and 2 963 from the COD. Although the vast majority of the structures in the MC3D-source are experimental, some of the structures extracted from the ICSD and COD were found to be flagged as theoretical, i.e. hypothesized in a theoretical study instead of being observed experimentally. Screening the metadata for these flags, we find 3 071 theoretical structures, so approximately 3.85% of the full structure set.

Due to licensing constraints, we are not allowed to publish the full MC3D-source structure set. Instead, we provide a YAML file on the Materials Cloud archive [10] called `MC3D_ids.yaml` that contains the list of versions and IDs for each structure extracted from the three databases.

Materials Project - MP

The Materials Project (MP) [11] dataset used contains a total of 83 989 bulk, crystalline, inorganic compounds that have been relaxed with first-principles calculations starting from experimental databases or from structure-prediction methods. It is retrieved through the Matminer [12] Python library. The version of the database employed in the study dates back to 10/18/2018, corresponding to the `p_all_20181018` dataset retrieved with the `matminer.datasets` module [13].

II. SOAP and FPS

In this study, it is necessary to have an ML representation that is invariant to symmetry operations and changes smoothly with the Cartesian coordinates. We choose Smooth Overlap of Atomic Positions (SOAP) vectors, a representation based on smoothed atomic densities: these are abstract feature vectors based on an expansion of atom-centered Gaussians in radial basis functions and spherical harmonics. This representation discretizes a three-body correlation function including information on each atom, its relationships with neighbouring atoms, and the relationships between sets of neighbours, quantifying similarities between atomic neighborhoods.

The 3-body SOAP vector is built as

$$\langle \alpha n \alpha' n' l | \mathcal{X} \rangle \propto \frac{1}{\sqrt{2l+1}} \sum_m \langle \alpha n l m | \mathcal{X} \rangle^* \langle \alpha' n' l m | \mathcal{X} \rangle \tag{1}$$

where α refers to the species of the considered atoms, and $\langle \alpha n l m | \mathcal{X} \rangle$ is the expansion of a density field over spherical harmonics and radial bases with n radial bases and l angular channels

$$\langle \alpha n l m | \mathcal{X} \rangle = \int d\mathbf{r} R_n(r) Y_m^l(\hat{\mathbf{r}}) \langle \alpha r | \mathcal{X} \rangle \tag{2}$$

Here $\langle \alpha r | \mathcal{X} \rangle$ encodes the species-tagged density field as a function of r with radius σ and accumulated until an interaction cutoff of r_{cut}. For the examples contained in the text, we have used $n_{max} = 4$, $l_{max} = 4$, $\sigma = 0.5$, and $r_{cut} = 3.5$. When using species-invariant SOAP vectors, we use the same hyperparameters but combine all species channels together.

When working with species-tagged SOAP vectors, in order to increase computational efficiency we select the most diverse features using a Furthest Point Sampling (FPS) algorithm, an unsupervised selection method which maximizes diversity (variance) of the selected vectors as measured by the mutual Euclidean distance.

III. PCovR: tuning the mixing parameter

The Principate Covariates Regression (PCovR) [14] combines the losses of Linear Ridge Regression (LRR) and Principal Component Analysis (PCA) through the mixing parameter β. The feature matrix which embeds the reduced SOAP representation is projected into latent space with an orthogonal projection. Finding the optimal projection to the latent space amounts to minimizing the loss, which happens when the projection is built out of the principal eigenvectors of the covariance matrix of the initial feature matrix.

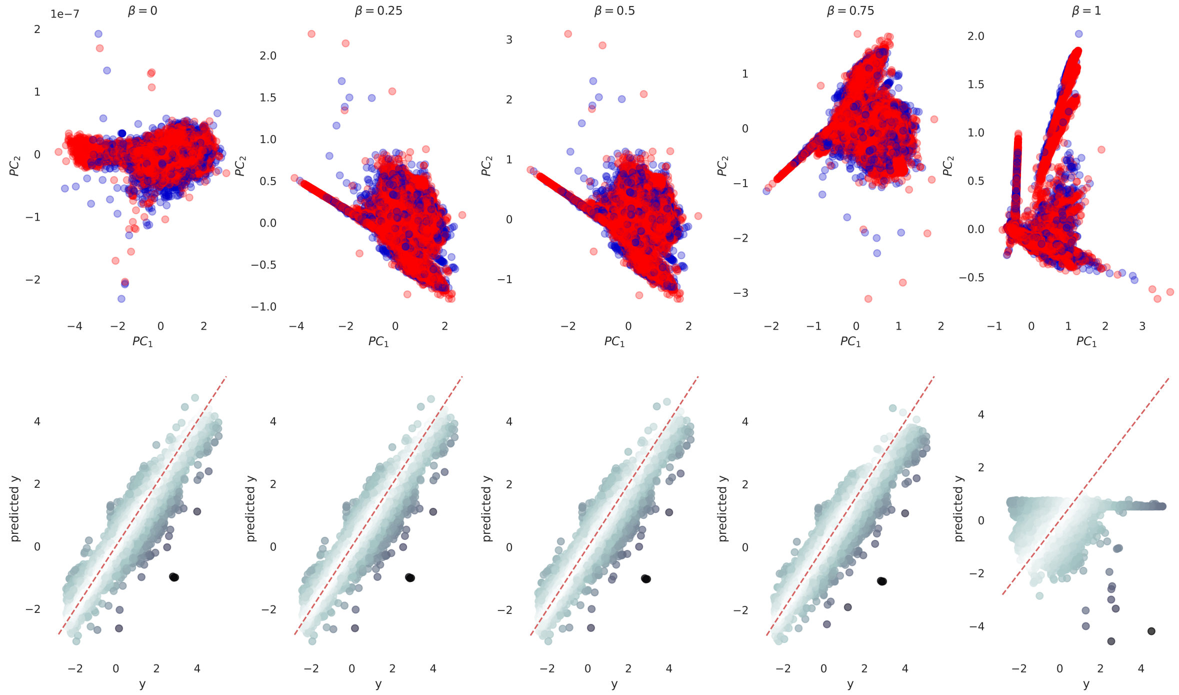

Supplementary Figure 1: The combination of LR (far left) and PCA (far right) in the PCovR analysis on the MP database. The resulting projections and regressions are shown at the indicated β values. RoF compounds are coloured in red, while non-RoF ones in blue.

IV. Energetic analysis with PCovR

The following section explores the PCovR energetic analysis performed on the MP dataset with the aim of classifying the structures into the two subgroups by performing a linear regression on local energetic descriptors only. Different covariates are plotted against the first principal covariate (on the x axis each time) to explore the full database variance. Each image is reported in two different views: on the left, the compounds are coloured according to their energetic property, i.e. formation energy per atom (Supplementary Figure 2), energy above the convex hull (Supplementary Figure 3) and band gap energy (Supplementary Figure 4), while on the right the same data is coloured according to the subset it belongs to using a kernel density probability estimation (KDE) normalised to the whole set of data. The isolated areas containing only RoF structures mostly contain structures with Mg-O square bonds, ionic bonds with high bond energy and therefore lower formation energy per atom. They validate the SOAP representation's usefulness in separating between compounds' subgroups, but are not enough to draw insightful conclusions on the RoF.

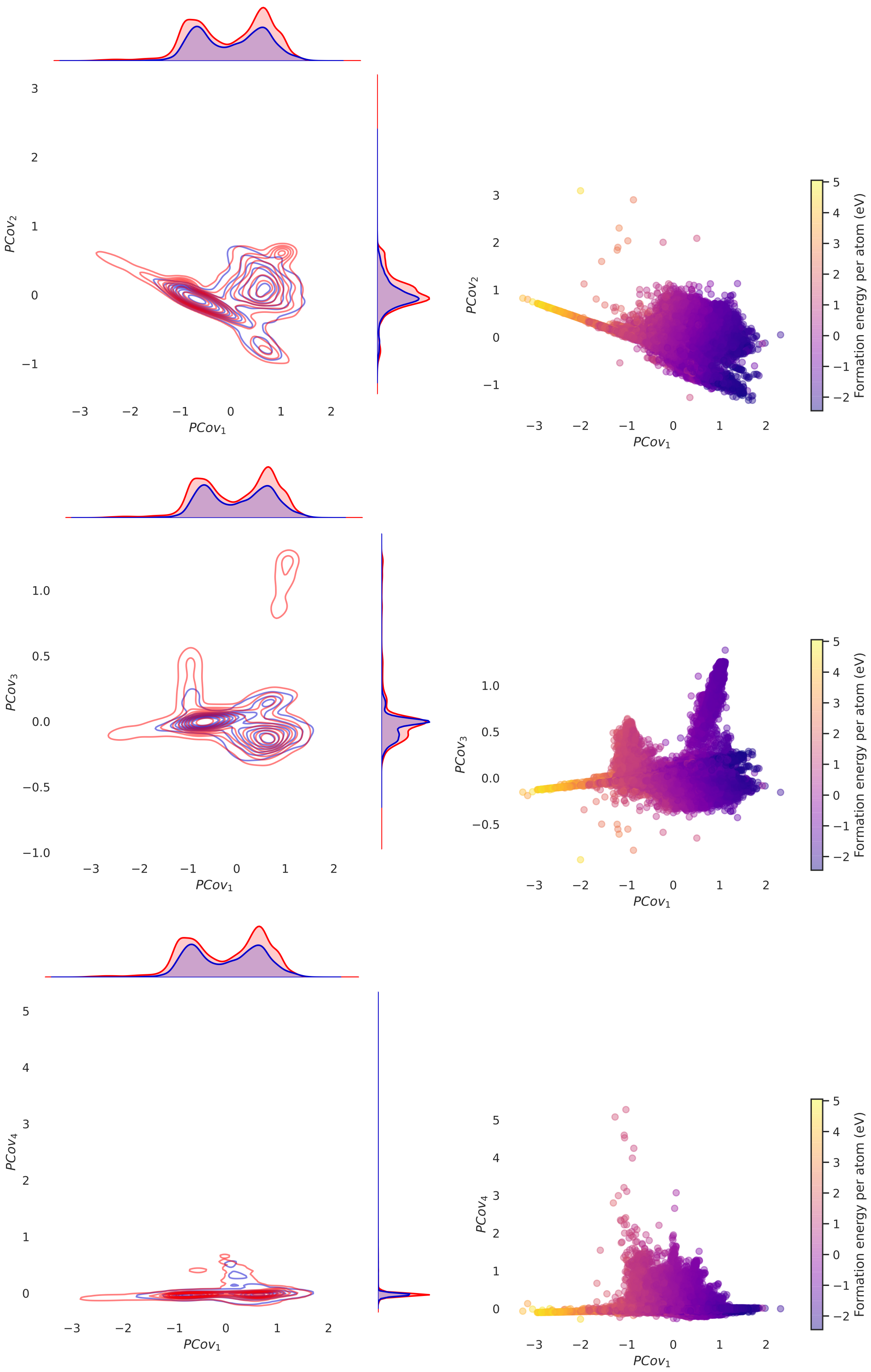

Supplementary Figure 2: PCovR representation with $\beta = 0.5$ containing information on SOAP representation and regressed on the formation energy per atom.

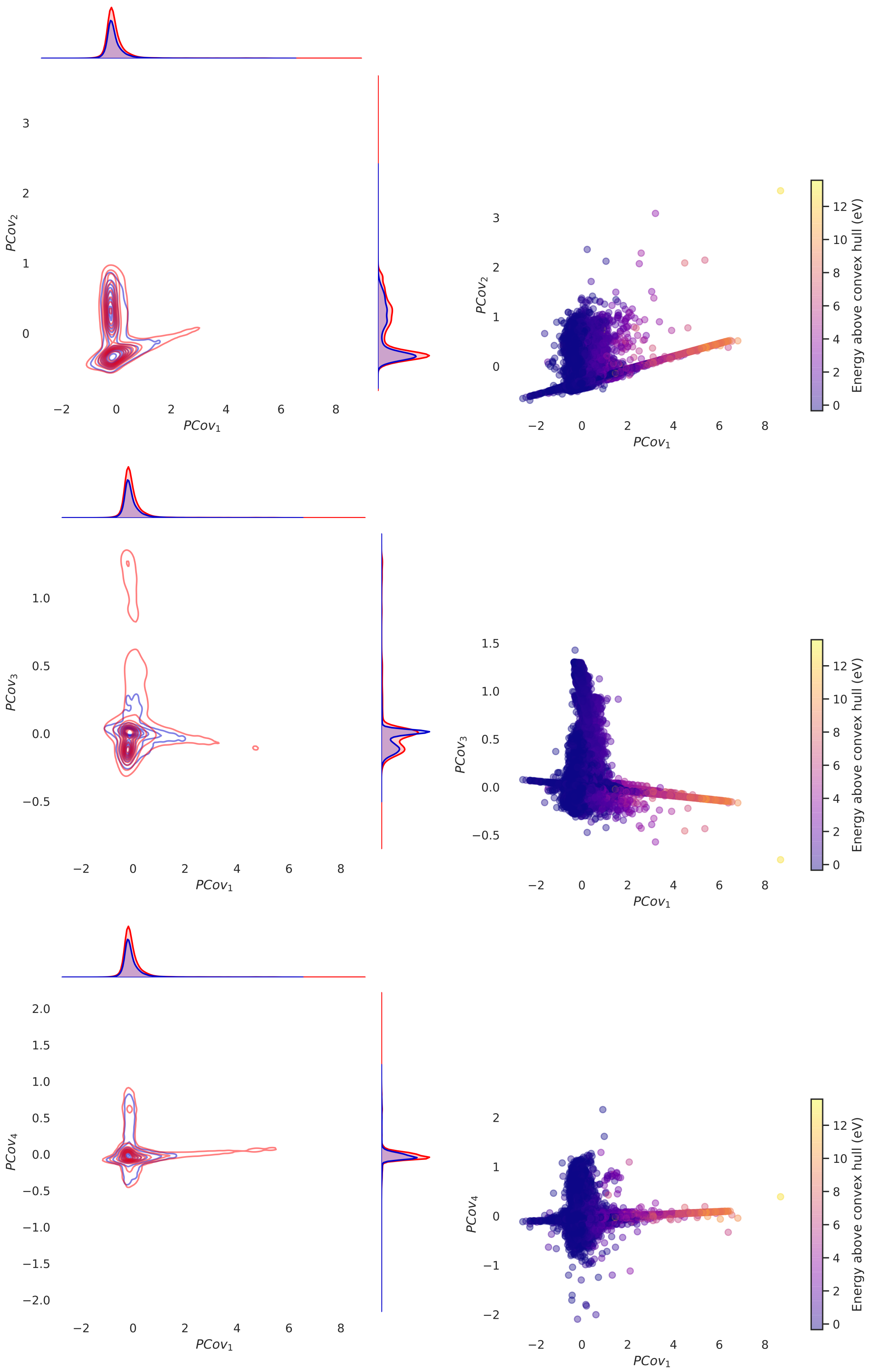

Supplementary Figure 3: PCovR representation with $\beta = 0.5$ containing information on SOAP representation and regressed on the energy above the convex hull.

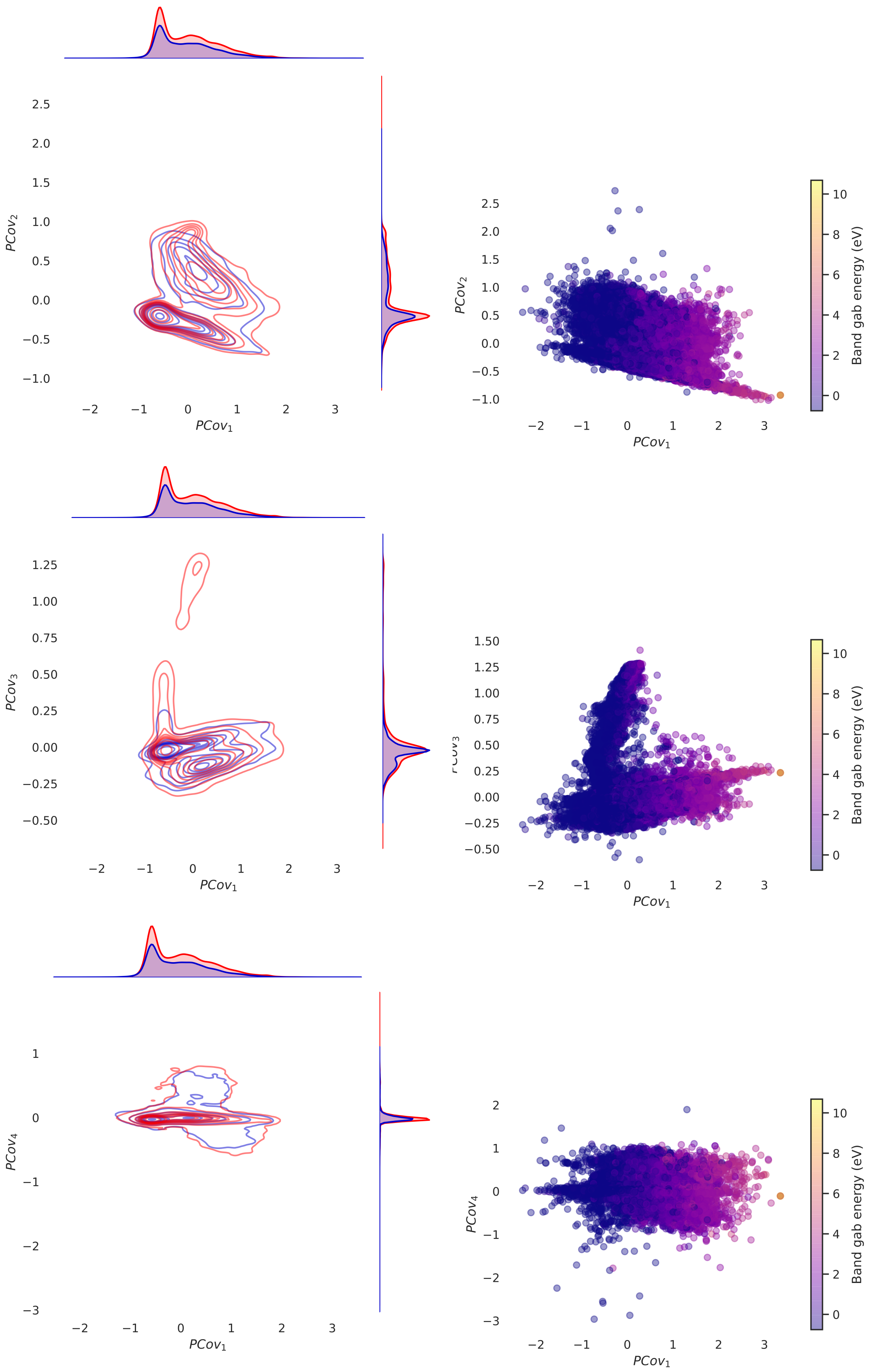

Supplementary Figure 4: PCovR representation with $\beta = 0.5$ containing information on SOAP representation and regressed on the band gap energy.

V. Classification algorithms

The statistics of the accuracy on the test set achieved by the different classification methods are reported in Supplementary Table 1. The Random Forest classifier [15] is not only the best-performing classifier, but has the smallest discrepancy between using species-invariant and species-tagged representations, which implies that the classification is primarily embedded in the local symmetries, despite the species information.

Classifier	Test Set R^2 (Species-Invariant)	Test Set R^2 (Species-Tagged)	Classifier parameters
Random Forest	0.871	0.88	{`bootstrap`: True, `ccp alpha`: 0.0, `class weight`: None, `criterion`: gini, `max depth`: None, `max features`: sqrt, `max leaf nodes`: None, `max samples`: None, `min impurity decrease`: 0.0, `min samples leaf`: 1, `min samples split`: 2, `min weight fraction leaf`: 0.0, `n estimators`: 100, `n jobs`: 4, `oob score`: False, `random state`: 2, `verbose`: 2, `warm start`: False}
MLP Classifier	0.728	0.801	{`activation`: relu, `alpha`: 0.0001, `batch size`: auto, `beta 1`: 0.9, `beta 2`: 0.999, `early stopping`: False, `epsilon`: 1e-08, `hidden layer sizes`: (100,), `learning rate`: constant, `learning rate init`: 0.001, `max fun`: 15000, `max iter`: 200, `momentum`: 0.9, `n iter no change`: 10, `nesterovs momentum`: True, `power t`: 0.5, `random state`: 2, `shuffle`: True, `solver`: adam, `tol`: 0.0001, `validation fraction`: 0.1, `verbose`: 2, `warm start`: False}
Decision Tree	0.587	0.805	{`ccp alpha`: 0.0, `class weight`: None, `criterion`: gini, `max depth`: None, `max features`: 80, `max leaf nodes`: None, `min impurity decrease`: 0.0, `min samples leaf`: 1, `min samples split`: 2, `min weight fraction leaf`: 0.0, `random state`: 2, `splitter`: best}
Linear SVM	0.594	0.67	{`C`: 1.0, `class weight`: None, `dual`: True, `fit intercept`: True, `intercept scaling`: 1, `loss`: squared hinge, `max iter`: 1000, `multi class`: ovr, `penalty`: l2, `random state`: 2, `tol`: 0.0001, `verbose`: 2}
Cross-Validated Logistic Regression	0.628	0.677	{`Cs`: 10, `class weight`: None, `cv`: 2, `dual`: False, `fit intercept`: True, `intercept scaling`: 1.0, `l1 ratios`: None, `max iter`: 100, `multi class`: auto, `n jobs`: 4, `penalty`: l2, `random state`: 2, `refit`: True, `scoring`: None, `solver`: lbfgs, `tol`: 0.0001, `verbose`: 2}
Stochastic Gradient Descent Classifier	0.594	0.61	{`alpha`: 0.0001, `average`: False, `class weight`: None, `early stopping`: False, `epsilon`: 0.1, `eta0`: 0.0, `fit intercept`: True, `l1 ratio`: 0.15, `learning rate`: optimal, `loss`: hinge, `max iter`: 100, `n iter no change`: 5, `n jobs`: 4, `penalty`: l2, `power t`: 0.5, `random state`: 2, `shuffle`: True, `tol`: 0.001, `validation fraction`: 0.1, `verbose`: 2, `warm start`: False}

Supplementary Table 1: Accuracy on test set achieved by different classifiers.

References

1. Huber, S. *et al.* Materials Cloud three-dimensional crystals database (MC3D). *Materials Cloud Archive.* https://archive.materialscloud.org/record/2022.38 (2022).
2. Giannozzi, P. *et al.* QUANTUM ESPRESSO: a modular and open-source software project for quantum simulations of materials. *J. Condens. Matter Phys.* **21,** 395502 (2009).
3. Giannozzi, P *et al.* Advanced capabilities for materials modelling with Quantum ESPRESSO. *J. Condens. Matter Phys.* **29,** 465901 (2017).
4. Pizzi, G., Togo, A. & Kozinsky, B. Provenance, workflows, and crystallographic tools in materials science: AiiDA, spglib, and seekpath. *MRS Bull.* **43,** 696–702 (2018).
5. Huber, S. P. *et al.* AiiDA 1.0, a scalable computational infrastructure for automated reproducible workflows and data provenance. en. *Sci. Data* **7,** 300. ISSN: 2052-4463. (2021) (2020).
6. Gražulis, S. *et al.* Crystallography Open Database – an open-access collection of crystal structures. *J. Appl. Crystallogr.* **42,** 726–729 (2009).
7. Levin, I. NIST Inorganic Crystal Structure Database (ICSD). *National Institute of Standards and Technology Public Data Repository.* https://doi.org/10.18434/M32147 (2018).
8. Villars, P., Cenzual, K., Gladyshevskii, R. & Iwata, S. The PAULING FILE Project and Materials Platform for Data Science: From Big Data Toward Materials Genome. *Springer, Cham.* https://doi.org/10.1007/978-3-319-42913-7_62-1 (2018).
9. Hinuma, Y., Pizzi, G., Kumagai, Y., Oba, F. & Tanaka, I. *Band structure diagram paths based on crystallography* 2016. arXiv: 1602.06402 [cond-mat.mtrl-sci]. https://arxiv.org/abs/1602.06402.
10. Gazzarrini, E., Cersonsky, R. K., Bercx, M., Adorf, C. S. & Marzari, N. The rule of four: anomalous stoichiometries of inorganic compounds. *Materials Cloud Archive.* https://archive.materialscloud.org/record/2023.104 (2023).
11. Jain, A. *et al.* Commentary: The Materials Project: A materials genome approach to accelerating materials innovation. *APL Mater.* **1,** 011002. (2021) (2013).
12. Ward, L. *et al.* Matminer: An open source toolkit for materials data mining. English (US). *Comput. Mater. Sci.* **152,** 60–69. ISSN: 0927-0256 (2018).
13. *Matminer, access ready made datasets in one line* https://hackingmaterials.lbl.gov/matminer/#access-ready-made-datasets-in-one-line. Accessed: 2023-07-24.
14. Helfrecht, B. A., Cersonsky, R. K., Fraux, G. & Ceriotti, M. Structure-property maps with Kernel principal covariates regression. en. *Mach. Learn.: Sci. Technol.* **1,** 045021. ISSN: 2632-2153. (2021) (Nov. 2020).
15. Breiman, L. Random Forests. English. *Mach. Learn.* **45,** 5–32. ISSN: 0885-6125 (2001).
